# Reassessing species demarcation criteria in viroid taxonomy by pairwise identity matrices

**DOI:** 10.1093/ve/veab001

**Published:** 2021-01-25

**Authors:** Michela Chiumenti, Beatriz Navarro, Thierry Candresse, Ricardo Flores, Francesco Di Serio

**Affiliations:** 1 Istituto per la Protezione Sostenibile delle Piante, Consiglio Nazionale delle Ricerche, Via Amendola 122/D, Bari 70126, Italy; 2 Univ. Bordeaux, INRAE, UMR BFP, Villenave d'Ornon Cedex, CS20032 33882, France; 3 Instituto de Biología Molecular y Celular de Plantas, Consejo Superior de Investigaciones Científicas–Universidad Politécnica de Valencia, Valencia 46022, Spain

**Keywords:** viroid classification, pairwise identity threshold, viroid species

## Abstract

With a small, circular and non-protein coding RNA genome, viroids are the smallest infectious agents. They invade plants, which in turn may develop symptoms. Since their discovery about 50 years ago, more than thirty viroids have been reported and classified using as species demarcation less than 90 per cent sequence identity on the overall genome and evidence of biological divergence with respect to the closest related viroids. In the last few years, new viroids have been identified that infect latently their (frequently) woody hosts and have a narrow experimental hosts range, complicating and slowing down studies on their biology. As a consequence, several viroids are still waiting for classification. Moreover, the number of new viroids is expected to increase in the next years due to the use of high-throughput sequencing technologies with diagnostics purposes. Therefore, establishment of reliable species demarcation criteria mainly based on molecular features of viroids is needed. Here, viroid classification is reassessed and a scheme based on pairwise sequence identity matrices is developed. After identifying a threshold pairwise identity score (PWIS) for each viroid genus, to be used as a species demarcation criterion, we show that most of those yet unclassified viroids can be assigned to a known or to a new species, thus limiting the need for additional biological evidence to only a few more complex situations. The advantages of this PWIS-based method are that the proposed identity thresholds for species demarcations are not arbitrarily established and evidence for biological divergence is not mandatory. Importantly, the current classification is not essentially modified. A protocol for a tentative fast classification of new viroids according to the proposed approach is also provided.

## 1. Introduction

Viroids are tiny, circular, single-stranded RNAs that, despite their lack of protein-coding capacity, infect higher plants (Flores et al. 2015; Adkar-Purushothama and Perreault 2020). About 50 years after their discovery (Diener 1971; Semancik and Weathers 1972), more than thirty viroids have been identified. Most of them are the causal agents of plant diseases with economic impact in agriculture ([Bibr veab001-B27][Bibr veab001-B29]; Rodriguez et al. 2017; Verhoeven et al. 2017). However, in several viroid–host combinations, no obvious symptoms are elicited (Skoric 2017). Based on biological, structural and biochemical features, viroids have been classified in two families: *Pospiviroidae* and *Avsunviroidae* (Di Serio et al. 2018a). Members of the family *Pospiviroidae* replicate in the nucleus, adopt rod-like or quasi rod-like conformations with conserved structural motifs [i.e. the central conserved region (CCR) involved in replication, and the terminal conserved region (TCR) or the terminal conserved hairpin (TCH)] that have taxonomic relevance to assign viroid species within five genera (Di Serio et al. 2020). Viroids in the family *Avsunviroidae* replicate and accumulate in plastids, assume rod-like or branched conformations, contain hammerhead ribozymes involved in replication and lack the CCR, TCR and TCH. The conformation of the genomic RNA and the G + C content, together with the structure of the hammerhead ribozymes, are the criteria used to assign the species within three genera in the family *Avsunviroidae* (Di Serio et al. 2018a). This classification scheme is consistent with the clustering in phylogenetic trees inferred from the genomic sequences of representative members of the recognized viroid species (Elena et al. 1991; Owens et al. 2012).

Similar to other RNA replicons, viroid populations from a single host are generally heterogeneous, being composed of closely related sequence variants slightly differing from each other. This sequence variability is likely due to the low fidelity of the DNA-dependent RNA polymerases involved in viroid replication (Flores et al. 2015) and confers to these infectious agents the typical features of quasispecies (Codoñer et al. 2006), a concept previously proposed for viruses (Biebricher and Eigen 2006; Andino and Domingo 2015). Besides implications in viroid evolution (Sardanyes et al. 2008), the quasispecies nature of these infectious agents is also related to the variability existing among populations from different isolates that may pose taxonomic issues, especially when novel viroid variants are to be assigned as members of a new or an established species.

The intrinsic variability of these infectious agents was taken into consideration since early attempts at classifying viroids (Flores 1995; Flores et al. 1998). Indeed, an arbitrary limit of less than 90 per cent sequence identity with other viroids over the entire genome was defined as the species demarcation threshold to develop the first viroid classification scheme (Flores et al. 2000). Such a sequence identity value appeared consistent with the divergent biological features shown by the known viroids. Therefore, different biological properties were initially only considered to solve some conflicting issues (Flores et al. 2005). A few years later, more stringent criteria were adopted by the ICTV. Indeed, experimental evidence of biological divergent features (i.e. host range, symptomatology, trafficking, vertical transmission) with respect to previously classified viroids, besides verification of the 90 per cent sequence divergence cut-off, became mandatory to create new or to reassess recognized species (Owens et al. 2012). Although these new criteria avoided the proliferation of novel viroid species, they were occasionally difficult to apply. This is the case for viroids with a restricted host range or that are symptomless in their natural hosts, such as apple fruit crinkle viroid (AFCVd, Ito et al. 1993), Coleus blumei viroid 5 (CbVd-5, Hou et al. 2009a), CbVd-6 (Hou et al. 2009b) and grapevine yellow speckle viroid 3 (GYSVd-3, Jiang et al. 2009), which were identified and proposed as potential new viroid species some years ago, but still remain as tentative species because discriminating biological features have not been determined (Di Serio, 2020). Moreover, the increasing use of high-throughput sequencing (HTS) technologies for diagnostics in plant virology (Hadidi et al. 2016) and the development of specific bioinformatics tools to search for circular RNAs from HTS data (Wu et al. 2012; Zhang et al. 2014) have allowed the identification of a range of additional new plant viroids or viroid-like RNAs in the last few years, primarily from non-symptomatic hosts. At the moment, in addition to the 32 recognized viroid species (Di Serio et al. 2018a; Di Serio, 2020) at least 12 new viroids are still waiting for proper classification (see below), mainly because their biological features have not been investigated or are difficult to address.

In viruses, sequence-based taxonomy schemes were initially proposed to classify members of the families *Potyviridae* (Adams et al. 2005) and *Geminiviridae* (Muhire et al. 2013; Varsani et al. 2014; Brown et al. 2015). Since then, several approaches to demarcate genera and species thresholds, taking into account the large number of viral genome sequences available in databases, have been developed. In this context, the programs VIRIDIC (Virus Intergenomic Distance Calculator, Moraru et al. 2020), PASC (PAirwise Sequence Comparison; Bào, Chetvernin, and Tatusova 2012), DEmARC (DivErsity pArtitioning by hieRarchical Clustering; Lauber and Gorbalenya 2012) and SDT (Sequence Demarcation Tool for Windows; Muhire et al. 2014) are based on pairwise identity matrices and have been developed or successfully used to refine existing virus classification schemes and propose new taxa (i.e.  (Coronaviridae Study Group of the International Committee on Taxonomy of Viruses, 2020) Bào et al. 2012; Kuhn et al. 2016; Bào et al. 2017; Varsani and Krupovic 2017; Käfer et al. 2019; Laenen et al. 2019). Although some of these methods could be extended to viroids, none of them specifically considers the peculiarity of viroid genomes, which are non-coding, small circular RNAs. Indeed, DEmARC was mainly designed for the analysis of conserved amino acid domains (Lauber and Gorbalenya 2012; Muhire et al. 2014). PASC compares the novel sequences in the configuration available in public sequence databases, complicating its application for circular RNAs, which occasionally have been deposited in databases with arbitrary termini. Moreover, PASC is mainly based on BLAST alignments to calculate pairwise matrices, an approach that does not seem appropriate for those viroids likely derived from recombination events between distantly-related ancestors, such as Australian grapevine viroid (AGVd) (Rezaian, 1990) , grapevine yellow speckle viroid 2 (Koltunow and Rezaian 1988), pear blister canker viroid (Hernández et al. 1992), columnea latent viroid (Hammond et al. 1989), dahlia latent viroid (Verhoeven et al. 2013) and citrus bark cracking viroid (CBCVd, Semancik and Vidalakis 2005), which are mosaics of sequences sharing identity with viroids from different genera. These limitations are not present in SDT, a method specifically developed to determine strain, species and genus demarcation thresholds based on pairwise identity that is not restricted to the use of predefined sets of sequences. However, akin to PASC, the calculation of pairwise identities in SDT ignores positions containing indel characters in the alignments. In the non-coding viroids genome, indels are very frequently observed between isolates, so that their complete exclusion does not seem appropriate for classification purposes because it would result in an overestimation of the pairwise identity scores.

Here, a reassessment of viroid classification is performed, with the specific aim of determining threshold demarcation criteria between species based on pairwise sequence identity matrices developed for the full-length genomic sequences available in NCBI databases. We show that the proposed sequence-based approach would allow the immediate allocation of all viroids yet unclassified to the current ICTV scheme. A protocol for a fast classification of novel viroids according to the proposed species demarcation criteria is also provided. This approach based on pairwise identity matrices would simplify the classification of the novel viroids expected to be identified by HTS in the future, limiting the need for biological supporting evidence to conflicting issues.

## 2. Methods

### 2.1 Origin of sequence variants and analyses of pairwise identity matrices

Members of viroid species were searched using ICTV approved names (https://talk.ictvonline.org/taxonomy/) in the NCBI nucleotide database (https://www.ncbi.nlm.nih.gov/nuccore) and their sequences downloaded in fasta format. For each viroid species, sequences annotated as partial genomes were discarded, while those corresponding to full-length genomes were aligned using MUSCLE (Edgar 2014). Alignments were manually checked for the presence of sequences starting at a different genomic coordinate, of partial sequences that had escaped the first cleaning step and of sequences with inconsistent changes in conserved regions/motifs, such as the CCR, TCR, TCH or in the nucleotide core of hammerhead ribozymes. These sequences were excluded from further analyses. Sequences obtained only by HTS approaches were also discarded. The cleaned-up fasta files including variants of each species, for a total of 3,959 viroid genomes, were aligned at species, genus and family level using MUSCLE (open gap penalty: 15; extended gap penalty: 6.66) embedded in the MacVector platform (version 15.1.5, Inc. Apex, North Carolina, USA). In preliminary tests, MUSCLE was found to be more conservative than CLUSTALW with the used large datasets, as also previously shown (Muhire et al. 2013; Varsani et al. 2014). Pairwise identity matrices were generated considering the indels in the alignments. A customized R script was run on all the obtained identity matrices to generate frequency distribution plots. Sequences used in this study are provided as supplementary material (see Supplementary fasta files).

### 2.2 Phylogenetic analyses

Reference sequences for each viroid species were aligned using ClustalW (gap opening and gap extension penalties 15 and 6.66, respectively) implemented in MEGA version X (Kumar et al. 2018). ClustalW was used because it provides close-to-optimal results, especially when sequences with diverse degrees of divergence are aligned (Higgins et al. 1994). The best substitution model was found to be the Tamura-Nei model (Tamura and Nei 1993) and the maximum likelihood method with 1,000 replicates was used to obtain a phylogenetic tree with the bootstrap values associated with each node.

## 3. Results and discussion

### 3.1 Pairwise identity matrices at family and genus levels

**Figure 1.**
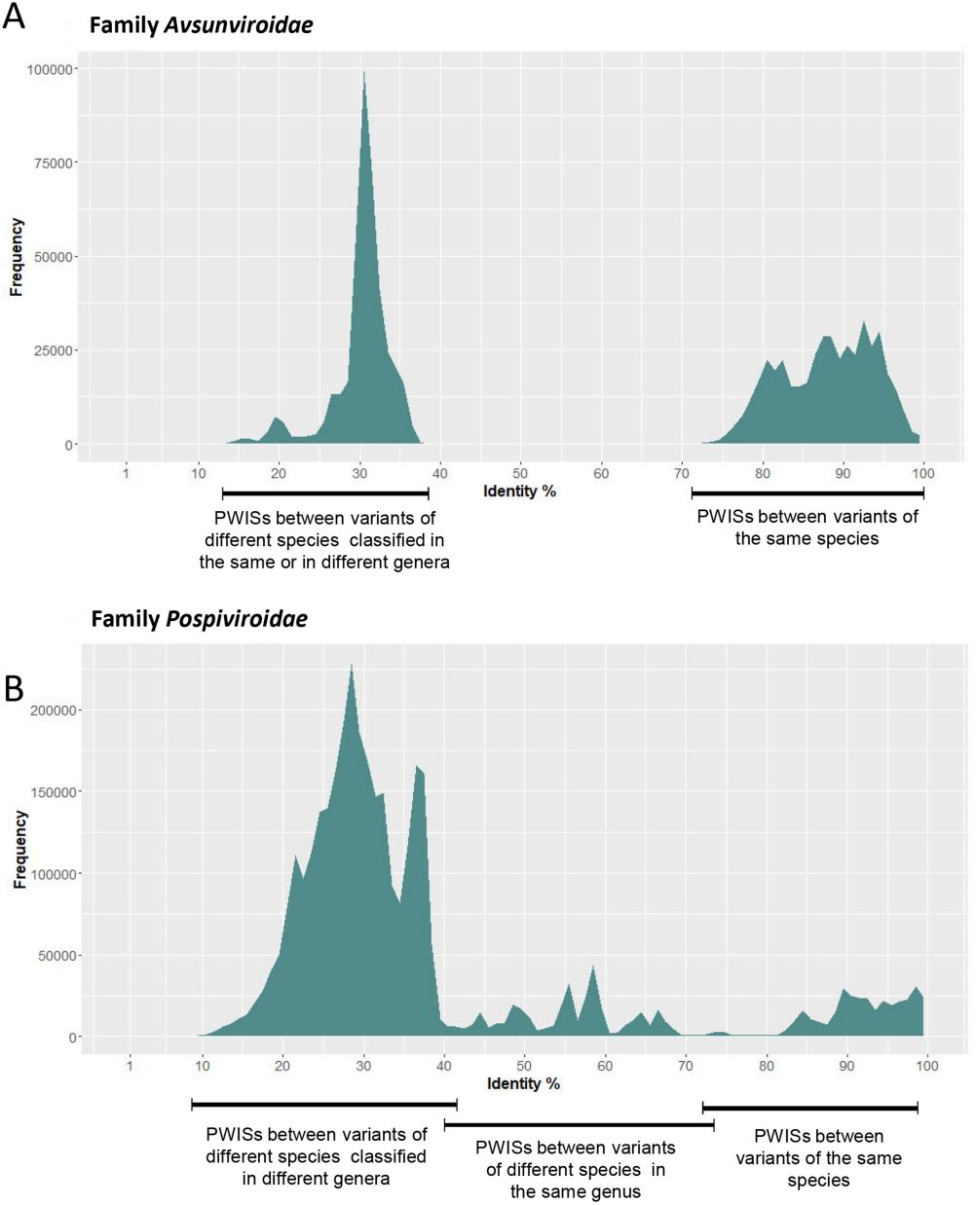
Distribution of PWISs between full-length viroid variants currently classified in the family *Avsunviroidae* (A) and *Pospiviroidae* (B). Frequencies of PWISs between variants classified in different genera, in different species of the same genera, and in the same species are indicated below the graphics.

A total of 3,959 full-length viroid genomic sequences, corresponding to variants classified according to the current ICTV species demarcation criteria (Di Serio et al. 2018a; Di Serio, 2020; Di Serio et al., 2020), were analyzed. Genomic sequences of viroids of the family *Avsunviroidae* and *Pospiviroidae* were treated separately. When pairwise identity scores (PWISs) were calculated (Supplementary Tables S1 and S2) and their frequencies plotted, two different distribution profiles were obtained (Fig. 1A). In the family *Avsunviroidae*, the PWISs formed two main peak groups, ranging from 15 per cent to 38 per cent and from 73 per cent to 100 per cent, respectively. The two groups were separated by a wide valley, the frequency of which was zero. In the family *Pospiviroidae*, a more continuous distribution of PWISs, with only two narrow valleys with frequency zero (ranging from 70% to 73% and from 76% to 81%), were observed (Fig. 1B). Deeper analyses of the two distribution profiles and of the original pairwise identity matrices showed that, as expected, the PWISs between variants classified in the same species had the highest values (Fig. 1). However, while in the family *Avsunviroidae* the lowest limit of pairwise sequence identity between variants belonging to the same species could be easily established at about 73 per cent (Fig. 1A), the situation appeared more complex in the *Pospiviroidae*, where such a limit was less clear because PWISs between variants of some species overlapped with those of different species in the same genus (Fig. 1B). Moreover, the PWISs between variants of viroids of different species belonging to the same genus generated frequency peaks from 40 per cent to 76 per cent in the family *Pospiviroidae* (Fig. 1B), contrasting with the situation in the *Avsunviroidae*, where no identity values in a similar range (38–70%) were observed, thus generating the wide valley in the distribution profile (Fig. 1A). This is a direct consequence of the PWISs between viroids belonging to different species in the genus *Pelamoviroid*, the only one including more than one species in the *Avsunviroidae*, which are very low (19–38%) and overlapping with those (10–37%) of the viroids from the other two genera in the family. This finding is not surprising because genus demarcation criteria adopted for the family *Avsunviroidae* in the current ICTV classification scheme are based on features related to the secondary structure adopted by the viroid genomic RNA, while the primary structure is not considered (Di Serio et al. 2018a). In contrast, genus demarcation criteria in the family *Pospiviroidae* include nucleotide sequences in conserved genomic regions (CCR, TCR and TCH) (Di Serio, 2020).

Altogether these data, besides confirming the convenience of treating separately members of both viroid families, show that viroid variants have been assigned to species despite having a sequence identity with the other variants in the same species lower than the recommended 90 per cent species cut-off. The lowest identity value observed is at 73 per cent in the family *Avsunviroidae*, and at a similar level in the *Pospiviroidae.*

**Figure 2.**
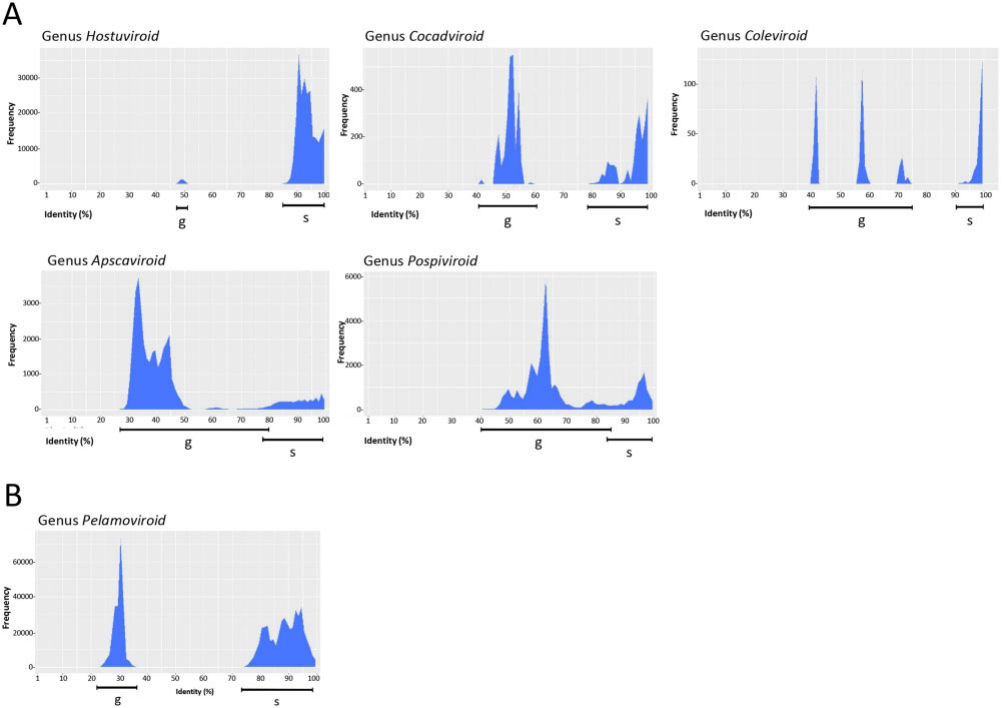
Distribution of pairwise identity scores among full-length viroid variants currently classified by ICTV in the genera *Hostuviroid, Cocadviroid. Coleviroid, Avsunviroid* and *Pospiviroid* of the family *Pospiviroidae* (A) and the genus *Pelamoviroid* of the family *Avsunviroidae* (B). The range of frequencies of PWISs between variants classified in different species (g) and in the same species (s) is indicated by horizontal lines below the graphics. The genera *Elaviroid* and *Avsunviroid* have not been included in the figure because being monotype genera, the distribution of PWISs at genus level are identical to those at species level (see Supplementary Fig. S1).

Matrices were then calculated for the genera including more than one species of the *Pospiviroidae* (Fig. 2A) and *Avsunviroidae* (Fig. 2B). The relative frequency distribution profiles of these matrices revealed that in most cases the highest PWISs were grouped in a single cluster corresponding to comparison values between variants of the same viroid species. Therefore, the lowest limit of such a cluster could mark the frontier between the variants of the same species and those of different species, thus establishing a threshold identity score (TIS) to be used as species demarcation criterion within each genus. With the exclusion of variants of genus *Coleviroid*, such a limit was always below the arbitrary limit of 90 per cent currently adopted by ICTV as one of the species demarcation criteria. Moreover, the peak distribution within the cluster of PWISs between variants of the same species was generally evenly distributed, without any wide valley with frequency zero. This feature indicates that the arbitrary sequence identity threshold of 90 per cent appears inappropriate as a TIS and that enforcing it as the only species demarcation criterion would artificially create novel species in several genera not well separated from the existing ones. Interestingly, the lowest limit of the PWIS cluster including variants of the same species largely differed among genera, ranging from 73 per cent to 90 per cent in the *Hostuviroid*, *Cocadviroid, Coleviroid* and *Pelamoviroid*. These results indicate that the intrinsic variability between variants of the same species is not uniform among viroid genera and that it appears convenient to incorporate these differences in the species demarcation criteria. For this reason, the identification of a TIS for each genus seems more appropriate than a single value established at the family or at a higher taxonomic rank. However, while in most genera, the PWISs between variants of the same species and those between variants of different species were largely separated by valleys of frequency zero (Fig. 2A and 2B), the PWISs between variants of the same and different species partially overlapped in the genera *Pospiviroid* and *Apscaviroid* (Fig. 2A), thus making more complex the identification of a potential TIS for them.

### 3.2 Determination of the threshold identity scores to be used as species demarcation criterion

**Figure 3.**
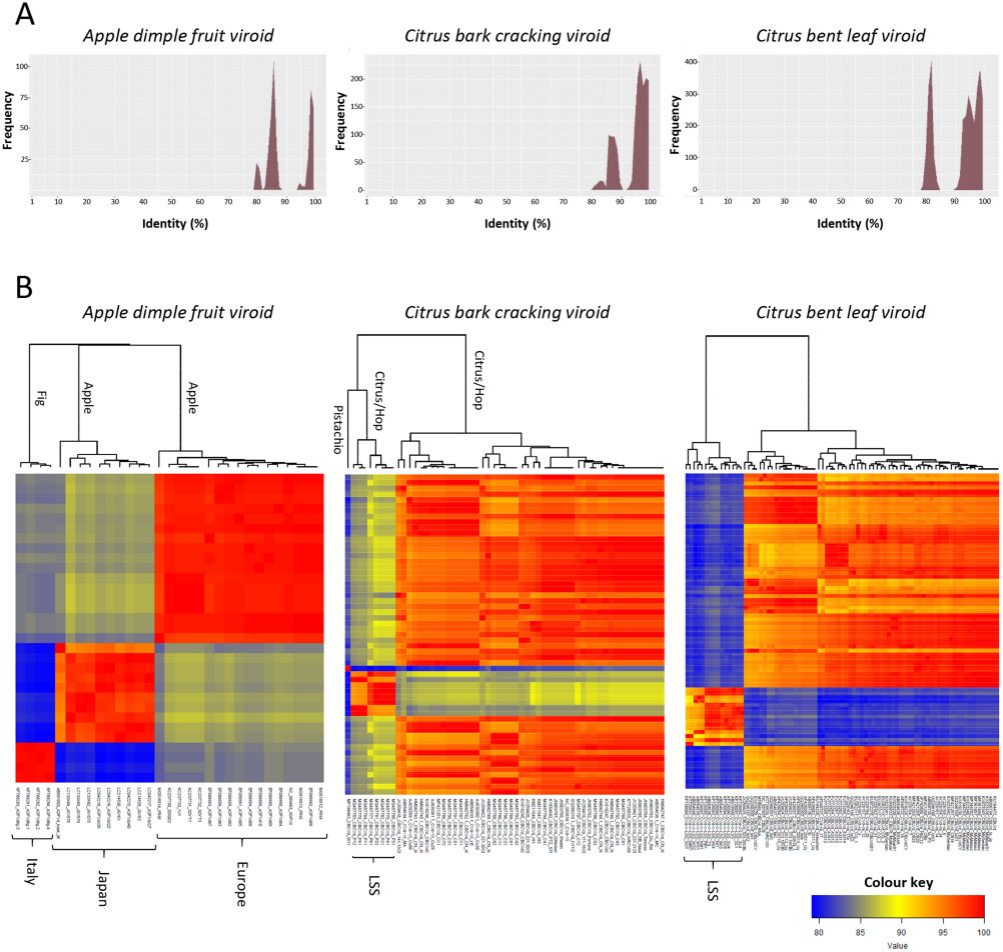
Distribution of PWISs (A) and two-dimensional pairwise identity color matrices (B) with pairwise identities calculated for the full-length viroid variants currently classified by ICTV in the species *Apple dimple fruit viroid*, *Citrus bark cracking viroid* and *Citrus bent leaf viroid*. The hierarchical dendrograms show clusters of variants with similar PWISs within each species.

Given the limitations reported above, pairwise identity matrices were calculated considering separately each viroid species. The values ranged from a variable lower limit (78.4–98.7%) to almost 100 per cent (Table 1) with frequency distribution profiles differing between species (Supplementary Fig. S1). In most species, a single cluster of values consisting of a single or a few frequency peak(s) without any frequency zero separating valleys was observed (Supplementary Fig. S1), indicating the existence of a homogenous group of variants. However, in ADFVd, CBLVd (genus *Apscaviroid*) and CBCVd (genus *Cocadviroid*), the PWISs were distributed forming two major clusters well separated by a valley with frequency zero (Fig. 3A), thus highlighting the existence of at least two major divergent populations of variants. Such diverging populations, which are also clearly visible by the color distribution in the pairwise identity matrices (Fig. 3B), raise the question of whether they should be considered as different species or as different strains within the same species. In ADFVd, the lowest pairwise values are between variants identified in apple (Di Serio et al. 1996) and in fig (Chiumenti et al. 2014), thus suggesting that sequence divergence is correlated with different natural hosts. In addition, variants infecting apple are also separated according to their geographic origin (Italy vs. Japan) (Fig. 3B), indicating the existence of at least two clearly distinct, divergent populations infecting the same host (Kasai et al. 2017). CBCVd has been reported in citrus, hop and, recently, pistachio (Al Rwahnih et al. 2018), with the variant infecting the latter host generating the lowest identity values when paired with the other ones (Fig. 3B). In contrast, variants infecting hop and citrus are not highly divergent from each other, although some from citrus isolates with higher sequence divergence, therefore denoted as low sequence similarity (LSS) variants, have been recently reported (Wang et al. 2018). LSS variants have been also identified in CBLVd (Ito et al. 2000). These three examples highlight situations in which PWISs analyses may help. The resulting uncertainties may require further biological testing.

**Table 1.** Minimum and the maximum PWISs between the sequence variants of each viroid species

Family	Genus	Species	Abbreviation	**Minimum PWIS**	**Maximum PWIS**	Nr of analyzed sequences
*Pospiviroidae*	*Pospiviroid*	*Potato spindle tuber viroid*	PSTVd	88.4	100	348
		*Citrus exocortis viroid*	CEVd	83.5	100	238
		*Chrysanthemum stunt viroid*	CSVd	91.9	100	140
		*Columnea latent viroid*	CLVd	85.2	100	80
		*Iresine viroid 1*	IrVd1	96.5	100	12
		*Pepper chat fruit viroid*	PCFVd	92.6	100	58
		*Tomato apical stunt viroid*	TASVd	84.2	100	32
		*Tomato chlorotic dwarf viroid*	TCDVd	95	100	16
		*Tomato planta macho viroid*	TPMVd	90.9	100	20
	*Hostuviroid*	*Hop stunt viroid*	HSVd	79.4	100	692
		*Dahlia latent viroid*	DLVd	100	100	5
	*Cocadviroid*	*Coconut cadang cadang viroid*	CCCVd	97.6	100	13
		*Coconut tinangaja viroid*	CTVd	100	100	1
		*Citrus bark craking viroid*	CBCVd	79.9	100	57
		*Hop latent viroid*	HLVd	96.9	100	25
	*Apscaviroid*	*Apple scar skin viroid*	ASSVd	87.7	100	99
		*Apple dimple fruit viroid*	ADFVd	79.1	100	31
		*Australian grapevine viroid*	AGVd	93.8	100	161
		*Citrus bent leaf viroid*	CBLVd	78.4	100	81
		*Citrus dwarfing viroid*	CDVd	85.2	100	206
		*Citrus viroid V*	CVd-V	88.8	100	40
		*Citrus viroid VI*	CVd-VI	90.4	100	25
		*Grapevine yellow speckle viroid 1*	GYSVd-1	81.5	100	157
		*Grapevine yellow speckle viroid 2*	GYSVd-2	95.6	100	12
		*Pear blister canker viroid*	PBCVd	87.1	100	65
	*Coleviroid*	*Coleus blumei viroid 1*	CbVd-1	96	100	20
		*Coleus blumei viroid 2*	CbVd-2	98.7	99.7	8
		*Coleus blumei viroid 3*	CbVd-3	91.8	100	7
*Avsunviroidae*	*Avsunviroid*	*Avocado sunblotch viroid*	ASBVd	92.4	100	59
	*Pelamoviroid*	*Peach latent mosaic viroid*	PLMVd	73.9	100	923
		*Chrysanthemum chlorotic mottle viroid*	CCMVd	91	100	170
		*Apple hammerhead viroid*	AHVd	80.3	99.8	61
	*Elaviroid*	*Eggplant latent viroid*	ELVd	83.9	100	98

The matrices calculated at the species level allowed the identification of the minimum PWIS (mPWIS) between variants of each species (Table 1). We reasoned that the lowest mPWIS within each genus could be an appropriate TIS to be used as a species demarcation criterion. Indeed, all the known pairs of variants generating a PWIS lower than such a threshold have been already classified in different species of the same genus. Therefore, any novel variant fulfilling the criteria to be classified in a genus and with PWIS lower than the TIS, should be classified in another novel species. Accordingly, rounding down to the first integer as in Table 1, TISs to be used as species demarcation criteria in the family *Pospiviroidae*, could be 78 per cent, 79 per cent, 83 per cent, 79 per cent and 91 per cent for genera *Apscaviroid, Hostuviroid, Pospiviroid, Cocadviroid* and *Coleviroid*, respectively (Table 1). In the family *Avsunviroidae*, the identified thresholds could be 92 per cent, 83 per cent and 73 per cent for genera *Avsunviroid, Elaviroid* and *Pelamoviroid*, respectively.

The TISs reported above have the advantage of not introducing any change in the current ICTV classification scheme, in agreement with the ICTV code which states that one of the principles of nomenclature should be to ‘aim for stability’. Implicitly, this agreement indicates that such a method, although based exclusively on nucleotide sequence comparisons, captures most of the biological diversity between known viroids considered in the current ICTV classification scheme.

### 3.3 Classification of novel viroids based on pairwise identity scores

As a further step in the reassessment of viroid classification, the identified thresholds were tested for assigning those viroids which, in the absence of the biological data currently requested by the ICTV, have remained unclassified so far. They are viroids or viroid-like RNAs discovered by conventional methods [AFCVd, CbVd-5 and CbVd-6, GYSVd-3, citrus viroid VII (CVd-VII), persimmon viroid (PVd) and Portulaca latent viroid (PLVd)] (Ito et al. 1993; Hou et al. 2009a,b; Jiang et al. 2009; Nakaune and Nakano 2008; Verhoeven et al. 2015; Chambers et al. 2018) or by HTS technologies [apple chlorotic fruit spot viroid (ACFSVd), dendrobium viroid (DVd), grapevine hammerhead viroid-like RNA (GHVd), grapevine latent viroid (GLVd), lychee viroid-like RNA (LVd), PVd-2 and plum viroid I (PlVd-I)] (Leichtfried et al. 2019; Yang 2020; Wu et al. 2012; Zhang et al. 2014; Jiang et al. 2017; Ito et al. 2013; Bester et al. 2020). The viroid nature has been verified for AFCVd, CbVd-5 and CBVd-6, CVd-VII, GLVd and DVd. In the case of ACFSVd, PVd, PVd-2, PlVd-1, infectivity by grafting has been verified, but their autonomous replication in the absence of any helper virus has not. Finally, GHVd and LVd are viroid-like RNAs structurally similar to members of the genus *Pelamoviroid* (family *Avsunviroidae*) and *Apscaviroid* (family *Pospiviroidae*), respectively (Wu et al. 2012; Jiang et al. 2017), but their infectivity has not yet been confirmed experimentally.

**Figure 4.**
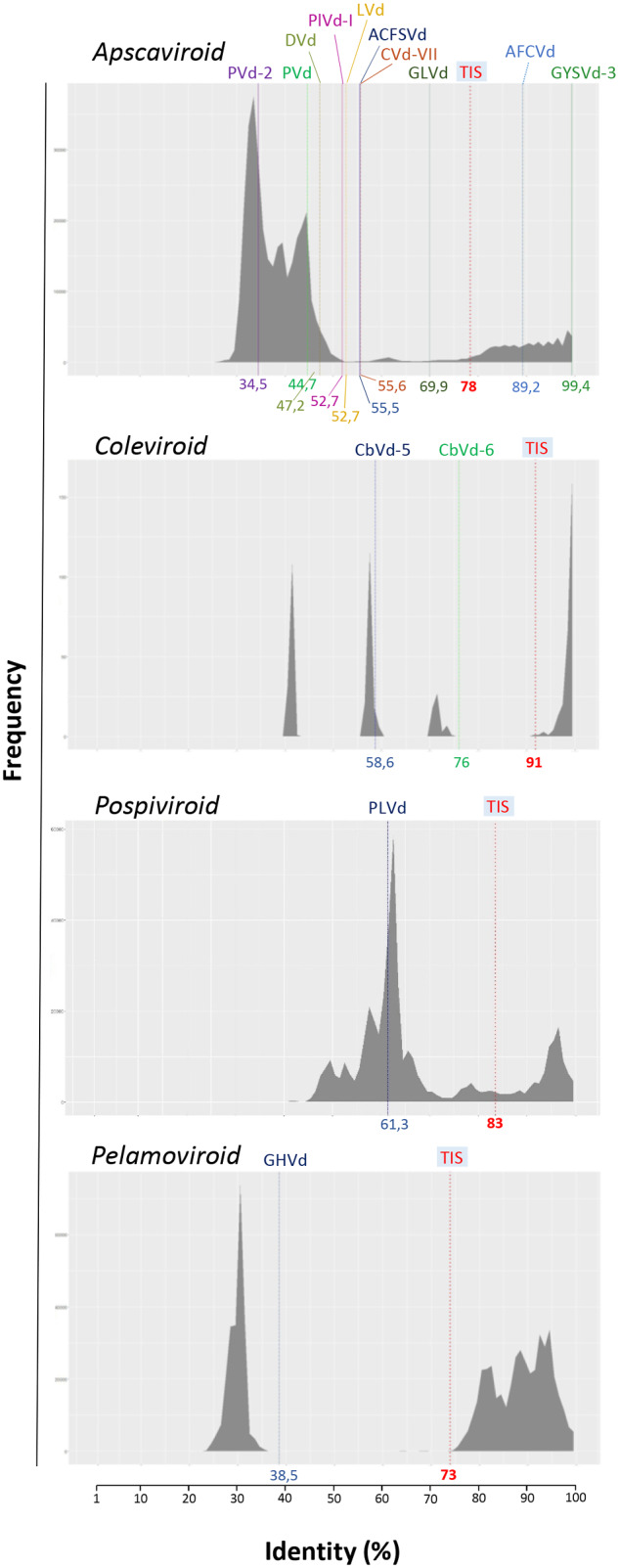
Distribution of PWISs between full-length sequence variants of viroids not classified yet of viroids currently classified in the genera *Apscaviroid, Coleviroid* and *Pospiviroid* of family *Pospiviroidae*, and in the genus *Pelamoviroid* of family *Avsunviroidae*. The TIS proposed as a species demarcation criterion for each genus is indicated below the graphic and marked by a broken, red, vertical line. The maximum PWIS between variants of each unclassified viroid is marked by a vertical line of different color for each viroid, with the numerical value (identity %) indicated below.

Since demarcation criteria for viroid genera do not include biological criteria (Di Serio et al. 2018a; Di Serio, 2020), all the unclassified viroids (or viroid-like RNAs) were tentatively assigned to a viroid genus in the original articles in which they were first described. With this information, pairwise identity matrices were calculated at the genus level, including in the alignments the variants of the unclassified viroids. In most cases, the maximum PWIS obtained for the variants of each novel viroid was consistently lower than the TIS for the genus to which they were tentatively assigned, thus indicating that the new viroids should be recognized as representing novel species (Fig. 4). Thus, ACLSVd, CVd-VII, GLVd, LVd, PVd and PVd-2 would become members of novel species in the genus *Apscaviroid*, while PLVd and CbVd-5 and CbVd-6 would be classified in novel species in the genera *Pospiviroid* and *Coleviroid*, respectively. If GHVd and LVd viroid-like RNAs are confirmed as genuine viroids, they should be classified as new species in the genera *Pelamoviroid* and *Apscaviroid*, respectively.

In contrast to these examples, variants of GYSVd-3 and AFCVd generated maximum PWIS (99.4% and 89.2%, respectively) higher than the TIS established for the genus *Apscaviroid* to which they were tentatively assigned (Fig. 4). A deeper analysis of the matrix showed that such maximum PWISs corresponded to GYSVd-3 and AFCVd variants paired with variants of GYSVd-1 and AGVd, respectively. These findings indicate that GYSVd-3 and AFCVd should be classified as variants of the *Grapevine yellow speckle viroid-1* and *Australian grapevine viroid* species, respectively. However, the two situations seem different. The maximum PWIS between GYSVd-3 and GYSVd-1 variants (99.4%), falls well within the range of PWISs already observed between GYSVd-1 variants (81.5–100%, Table 1), supporting its conclusive classification. In contrast, the maximum PWIS between AFCVd and AGVd variants (89.4%) is lower than the minimum PWIS calculated for AGVd variants (93.8%, Table 1), highlighting a slightly higher divergence between AFCVd and AGVd variants than previously identified in the AGVd variability landscape. AFCVd has been reported in apple and persimmon (Suzuki et al. 2017), but not in grapevine, which is the only known natural host of AGVd, thus generating some uncertainty on the convenience of classifying these viroids in the same species, and suggesting that additional biological data (i.e. host range of AFCVd and AGVd) should be considered. These examples, together with those regarding ADFVd, CBCVd and CBLVd already addressed above (see Section 3.2), illustrate that if even pairwise identity matrices may not solve all classification issues, they restrict the need of biological features to a few cases.

Uncertain situations may arise mainly when the PWIS calculated at the genus level for a new variant is lower than the minimum PWIS calculated for the closest related species, as exemplified by AFCVd, and/or when the PWIS of the novel unclassified variant is very close to the threshold identified at the genus level. The existence of such conflicting situations should not be surprising because natural variation among biological entities is continuous rather than discrete as requested by any classification scheme. Conflicting situations have also arisen in the current ICTV classification, where biological features are mandatory, as exemplified by the former species *Mexican papita viroid* that was removed after a reassessment, with variants of this viroid reclassified as members of the species *Tomato planta macho viroid* (Verhoeven et al. 2011).

**Figure 5.**
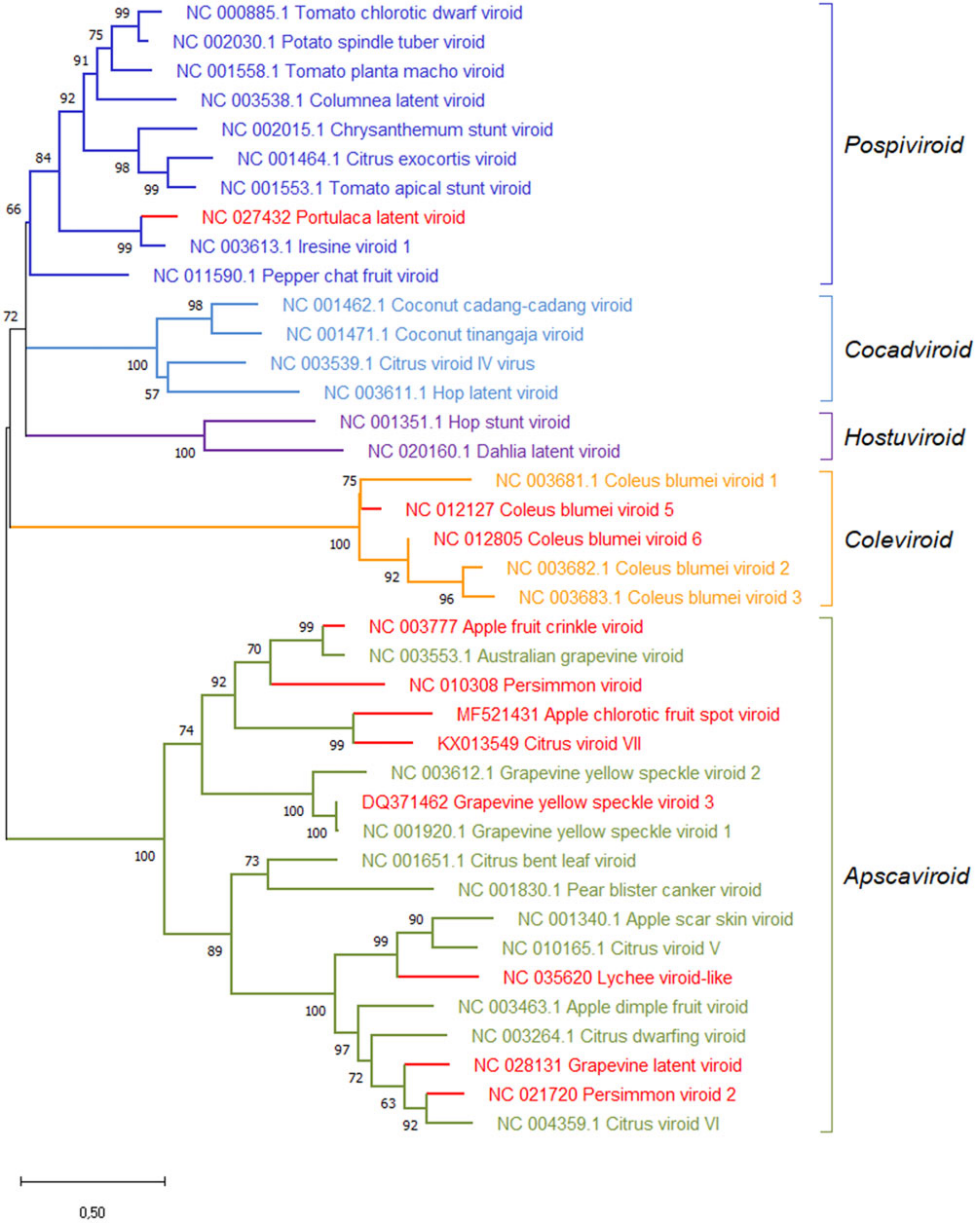
Maximum likelihood phylogenetic tree inferred with the reference variants of the species currently classified by ICTV in the five genera of the family *Pospiviroidae* and of the viroids yet unclassified (in red). Bootstrap values >70 per cent (generated by 1,000 replicates) are shown next to the branches. The tree is drawn to scale, with branch lengths measured in the number of substitutions per site.

The proposed novel viroid species in the family *Pospiviroidae* are also consistent with the clustering, the bootstrap values and the genetic distances observed in the maximum likelihood phylogenetic tree calculated for the reference sequence variants of the novel viroids and of all the species already classified in the family *Pospiviroidae* (Fig. 5).

### 3.4 Development of a fast protocol for classifying novel viroids

The identification of reliable pairwise identity thresholds and the classification attempts reported above requested deep analyses and time-consuming calculations of matrices using hundreds of variants. However, to be applicable at a larger scale, the method needs to be simplified. Therefore, we tested an alternative approach based on the generation of matrices using only the best BLAST-matching variants of a potential novel viroid. Similar approaches have been previously proposed for the classification of begomoviruses (Brown et al. 2015), curtoviruses (Varsani et al. 2014) and mastreviruses (Muhire et al. 2013).

**Figure 6.**
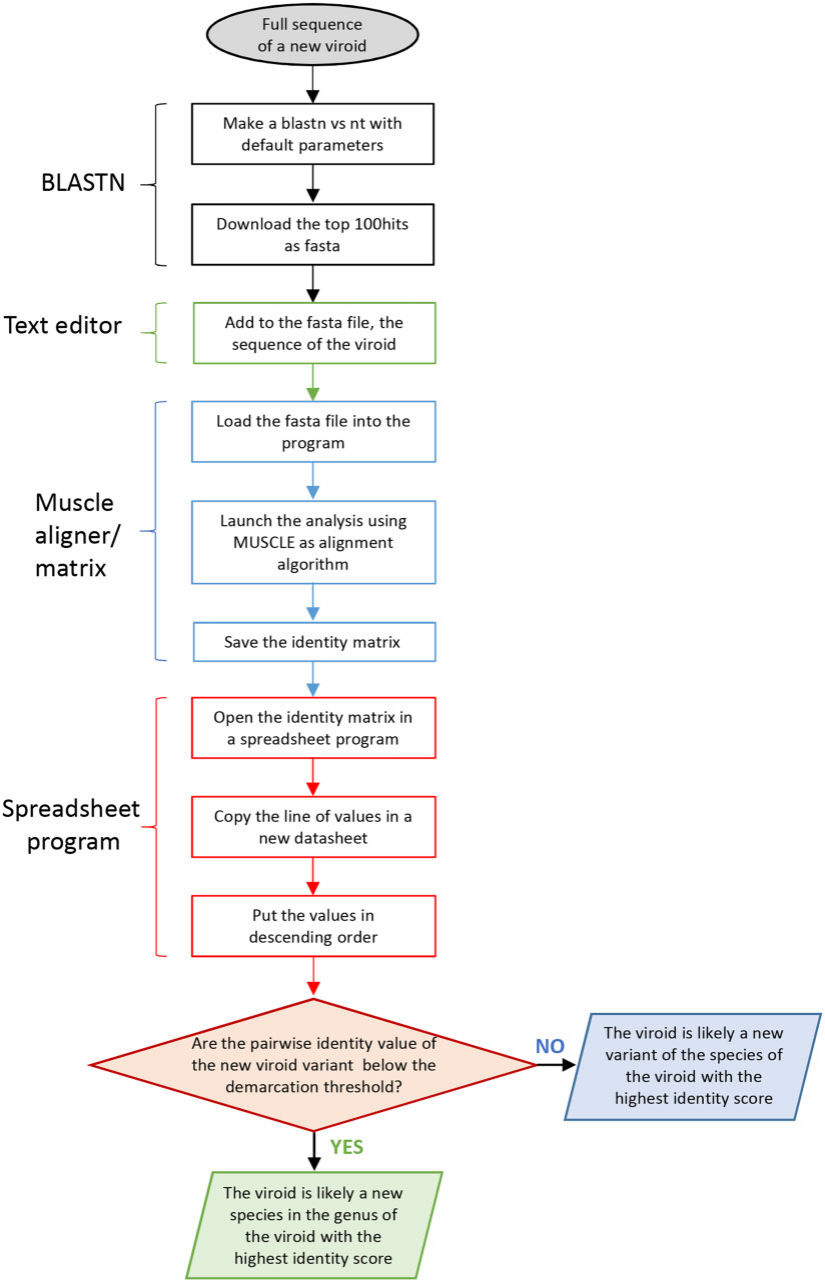
Flowchart of the main steps of a fast method proposed to classify novel viroids based on pairwise identity scores.

This faster method involves the following steps (Fig. 6):

1. A BLASTn analysis of the putative new viroid sequences is run against the ‘non-redundant nucleotide’ database (https://blast.ncbi.nlm.nih.gov/Blast.cgi) to identify viroid sequences with the highest identity to the new sequences. The top 100 hits are downloaded from the output page as a fasta file [Download → fasta (Complete sequence)].
2. The new viroid sequence(s) is (are) added to the downloaded fasta file using a text editor.
3. The modified fasta file is loaded into a sequence aligner program, and the sequences are aligned using MUSCLE without deleting the gaps for pairwise matrices computations.
4. Once the pairwise identity matrix has been calculated, the file is opened in a spreadsheet program (i.e. Microsoft Excel) and the values for the new species are displayed in descending order according to the maximum identity value with the variants already annotated in the database.
5. Attention is focused on the species and genus in which the viroid variants associated with the maximum PWISs are classified, to determine the relevant genus so that its demarcation threshold could be considered in the next comparative step.
6. If the maximum PWIS in the matrix is below the demarcation threshold established for the pertinent genus, the viroid variant could be regarded as a representative member of a new species to be created; otherwise, the new variants should be classified as members of the species showing the highest PWISs. However, especially if the maximum PWIS is very close to the TIS, further confirmation by considering the maximum PWIS obtained from a matrix calculated including as much as possible variants from the pertinent genus would be needed.

When this approach was applied to the unclassified viroids, the obtained maximum PWISs were in general higher than those calculated by considering all the variants of the species classified in each genus (Table 2). Although deviations between the two methods of up to 15–20 per cent in the case of PLVd, PlVd-I and PVd-2 were observed, PWISs values calculated with the fast method were still in agreement with the classification proposal outlined above (see Section 3.3). Therefore, the simplified approach based on the use of only the first 100 best matching BLAST sequences may not be accurate for some viroids, but still seems practicable for the tentative classification of new viroids, especially when the PWISs are quite far from the established thresholds.

**Table 2.** Maximum PWIS calculated for each viroid and viroid-like RNAs not classified yet by considering all the variants in their pertinent genus or the top 100 viroid sequences retrieved by BLAST

Genus	TIS (%)	Viroid names	Abbreviation	Maximum PWIS considering all variants in the genus	Maximum PWIS considering the top BLASTN hits
*Apscaviroid*	78	Apple chlorotic fruit spot viroid	ACFSVd	55.5	52.6
		Apple fruit crinkle viroid	AFCVd	89.2	89.3
		Citrus viroid VII	CVd-VII	55.6	54.1
		Dendrobium viroid	DVd	47.2	53.9
		Grapevine latent viroid	GLVd	69.9	71.1
		Grapevine yellow speckle viroid 3	GYSVd-3	99.4	99.5
		Lychee viroid	LVd	52.7	59.1
		Persimmon viroid	PVd	44.7	53.2
		Persimmon viroid 2	PVd-2	34.5	54.8
		Plum viroid I	PlVd-I	52.7	71.2
*Coleviroid*	91	Coleus blumei viroid 5	CbVd-5	58.6	68.5
		Coleus blumei viroid 6	CbVd-6	76	76.3
***Pospiviroid***	83	Portulaca latent viroid	PLVd	61.3	77
***Pelamoviroid***	73	Grapevine hammerhead viroid	GHVd	38.5	–

The proposed TIS for each genus is also indicated.

The SDT (SDTv1.2) method to calculate matrices is available online (http://web.cbio.uct.ac.za/∼brejnev/) and has the advantage of being fast and easy to use. However, since SDT does not take into consideration the indels in the alignments, PWISs in the SDT matrices are expected to differ from those calculated by the method proposed here, which does take into account the indels. Since the thresholds we propose here are based on matrices calculated without removing the indels in the alignments, the PWISs obtained by SDT are not expected to be fully comparable with the thresholds here proposed.

## Concluding remarks

With respect to the species demarcation criteria adopted in the current ICTV viroid classification, our proposed alternative based on PWISs and appropriate TISs has the advantage of not being dependent on the need of biological divergence between existing and new viroids to create novel viroid species. It is now possible to assess for classification most yet unclassified viroids, including those from asymptomatic and/or woody hosts or that have a narrow host range. Therefore, the pairwise identity-based method provides an effective option to decide whether a new viroid should be considered as representing or not a new species under most situations, including new viroids identified by HTS technologies. The TISs proposed in this study are not as arbitrary, as the limit of 90 per cent sequence identity used as species demarcation criterion in the current ICTV classification (Owens et al. 2012) but derived from pairwise identity matrices using the viroid sequence variants currently available in databases. The proposed TISs may change over time as our knowledge of the diversity of viroid species progresses. The threshold adopted for each genus could be regularly tested using updated databases and adjusted when needed, thus providing an adaptable system. Moreover, since the TISs correspond to values falling in a ‘valley? of the frequency distribution profiles of the respective genera (Fig. 4), they are expected to generate minimal conflicts (Brown et al. 2015). Indeed, the likelihood that a variant with a PWIS falling in a valley could be assigned to a known species is minimal. In addition, the more distant are the PWISs from such a threshold, the lower the uncertainties are associated when creating novel species for classifying divergent variants.

The pairwise identity matrices highlight the existence of strongly diverging populations of variants (e.g. for ADFVd, CBLVd, CBCVd and AGVd) in some established viroid species, thus opening the question of whether some species in the current ICTV classification framework need to be re-evaluated. Conflicting situations generated by the application of the proposed classification method have been also identified, for example when the PWISs calculated at the genus level for variants under test are lower than the minimum PWIS calculated for the closest related species, as exemplified by AFCVd, and/or when they are close to the threshold identified at the genus level. In all these cases, biological data may help to solve the uncertainties. Therefore, our proposed classification scheme does not dismiss biological data for classification purposes, but tries to reduce their relevance to only a few conflicting cases. Biological data needed to solve critical issues should therefore be established on a case by case basis, considering the specific taxonomic problem to address.

It is important to stress that the proposed method for viroid classification cannot replace the bioassays needed to ascertain the viroid nature of a new circular RNA (Di Serio et al. 2018b). This point is particularly relevant especially for viroid-like RNAs containing hammerhead ribozymes (Navarro et al. 2017), such as GHVd, for which the possibility that they could be viroid-like satellite RNAs should not be dismissed till bioassays will support their infectivity in the absence of any helper virus.

## Supplementary data

Supplementary data are available at *Virus Evolution* online.

## Supplementary Material

veab001_Supplementary_Data
