# Supplementary material for: Reassessing species demarcation criteria in viroid taxonomy by pairwise identity matrices
Source: Virus Evol. 2021 Jan 25;7(1):veab001. doi: 10.1093/ve/veab001 (PMC7887442; doi:10.1093/ve/veab001)

Figure S1 - Distribution of pairwise identity scores (PWISs) among full-length sequence variants of viroid species within the families *Avsunviroidae* (A) and *Pospiviroidae* (B) according with the current classification (https://talk.ictvonline.org/taxonomy/).

A

Genus *Avsunviroid*

Avocado sunblotch viroid

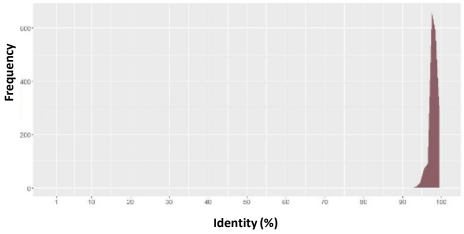

Genus *Pelamoviroid*

Peach latent mosaic viroid

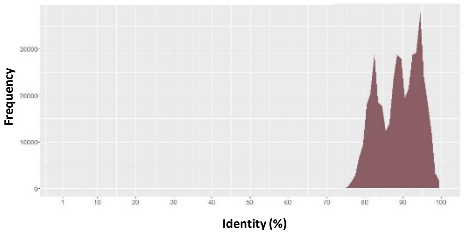

Chrysanthemum chlorotic mottle viroid

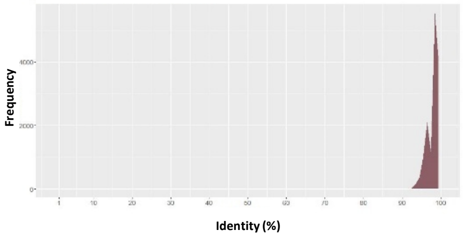

Apple hammerhead viroid

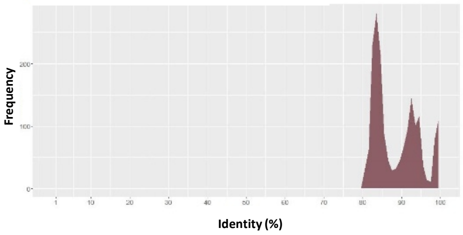

Genus *Elaviroid*

Eggplant latent viroid

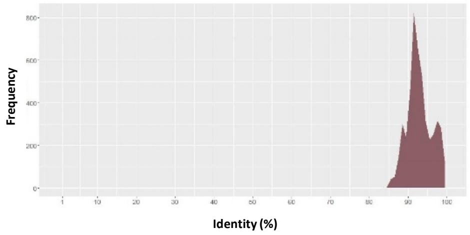

B

Genus *Pospiviroid*

Potato spindle tuber viroid

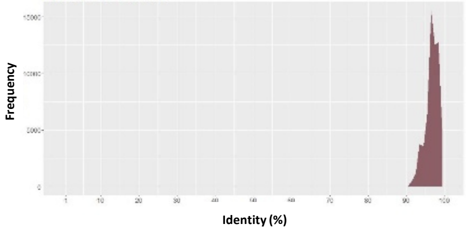

Citrus exocortis viroid

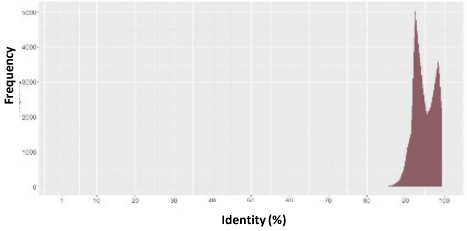

Chrysanthemum stunt viroid

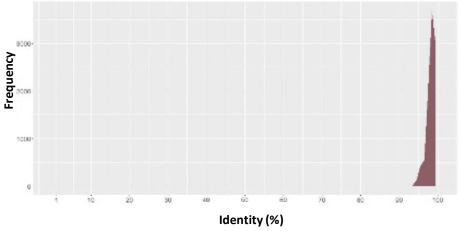

Columnea latent viroid

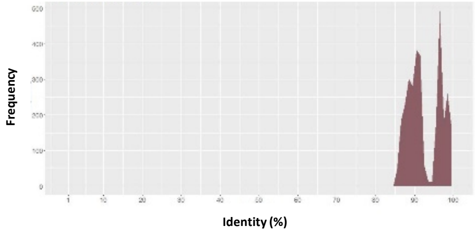

Iresine viroid 1

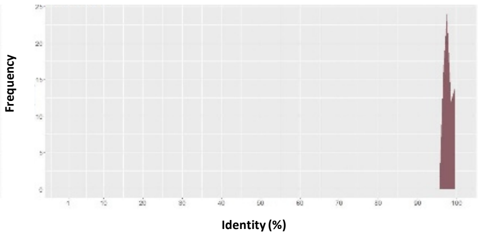

Pepper chat viroid

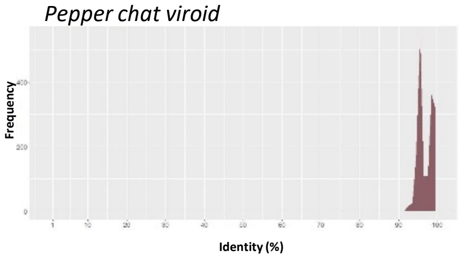

Tonato apical stunt viroid

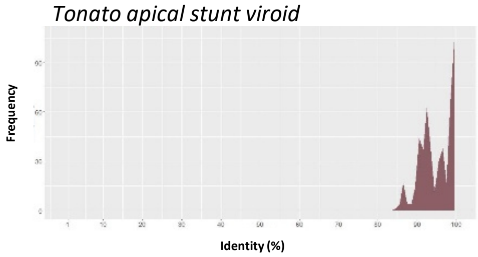

Tomato chlorotic dwarf viroid

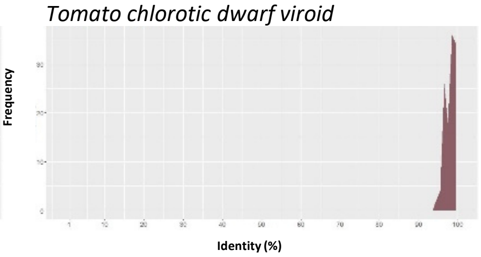

Tomato planta macho viroid

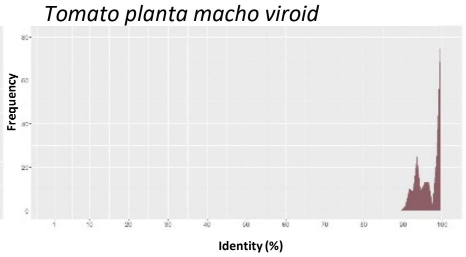

Genus *Hostuviroid*

Hop stunt viroid

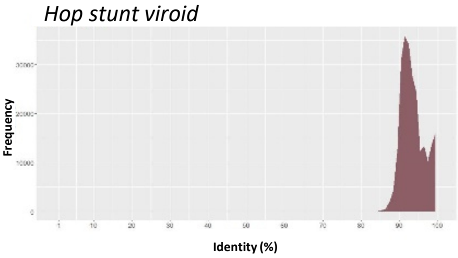

Dahlia latent viroid

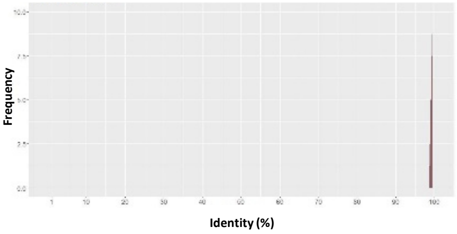

Genus *Cocadviroid*

Coconut cadang cadang viroid

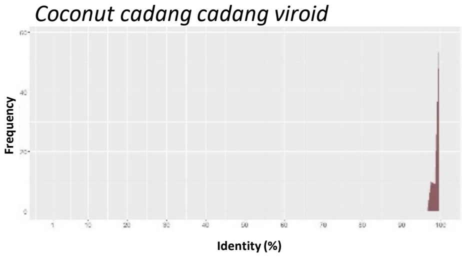

Hop latent viroid

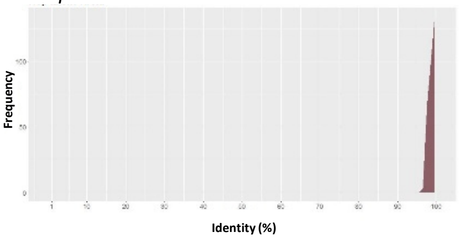

Genus *Apscaviroid*

Apple scar skin viroid

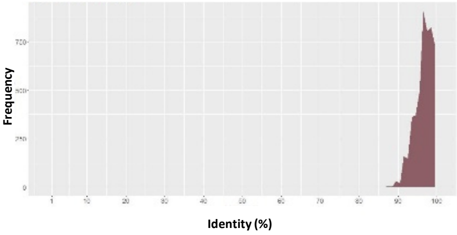

Australian grapevine viroid

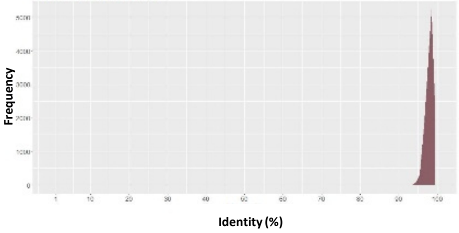

Citrus dwarfing viroid

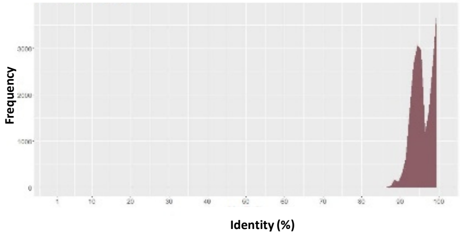

Citrus viroid V

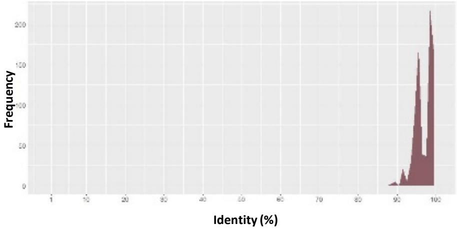

Citrus viroid VI

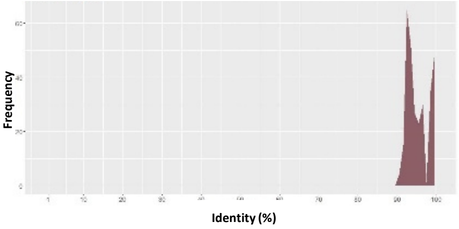

Grapevine yellow speckle viroid 1

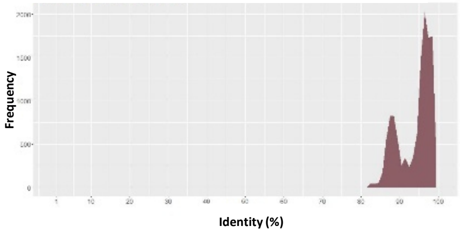

Grapevine yellow speckle viroid 2

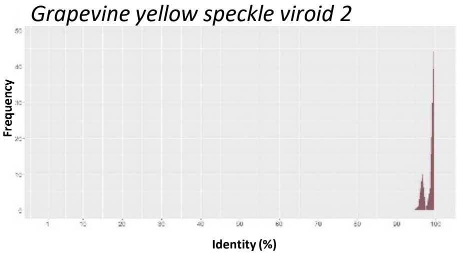

Pear blister canker viroid

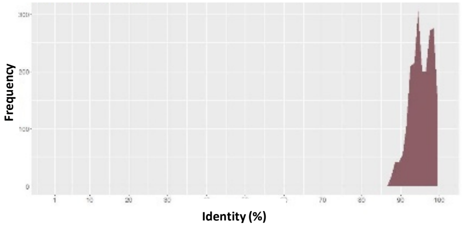

Genus *Coleviroid*

Coleus blumei viroid 1

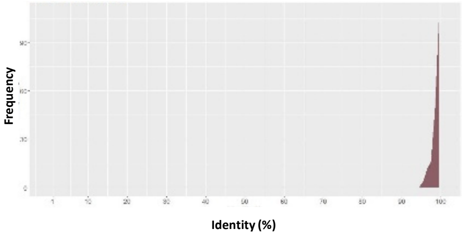

Coleus blumei viroid 2

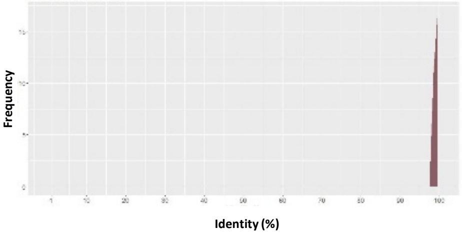

Coleus blumei viroid 3

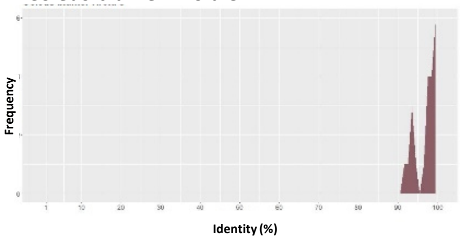

Supplement: veab001_Supplementary_Data [file veab001_supplementary_data.zip › Figure_S1 R2.pdf]
